# Modular Neural Mechanisms for Gait Phase Tracking, Prediction, and Selection in Personalizable Knee-Ankle-Foot-Orthoses

**DOI:** 10.3389/fnbot.2018.00037

**Published:** 2018-07-25

**Authors:** Jan-Matthias Braun, Florentin Wörgötter, Poramate Manoonpong

**Affiliations:** ^1^Computational Neuroscience Group, 3. Physics Institute, Georg-August-University, Göttingen, Germany; ^2^Bernstein Focus Neurotechnology, Georg-August-University, Göttingen, Germany; ^3^CBR Embodied AI & Neurorobotics Lab, The Maersk Mc-Kinney Moller Institute, University of Southern Denmark, Odense, Denmark; ^4^Bio-inspired Robotics & Neural Engineering Lab, School of Information Science & Technology, Vidyasirimedhi Institute of Science & Technology, Rayong, Thailand

**Keywords:** artificial neural network, neural orthosis control, adaptation, gait classification, level walking, stair climbing, internal model, model invalidation

## Abstract

Orthoses for the lower limbs support patients to perform movements that they could not perform on their own. In traditional devices, generic gait models for a limited set of supported movements restrict the patients mobility and device acceptance. To overcome such limitations, we propose a modular neural control approach with user feedback for personalizable Knee-Ankle-Foot-Orthoses (KAFO). The modular controller consists of two main neural components: neural orthosis control for gait phase tracking and neural internal models for gait prediction and selection. A user interface providing online feedback allows the user to shape the control output that adjusts the knee damping parameter of a KAFO. The accuracy and robustness of the control approach were investigated in different conditions including walking on flat ground and descending stairs as well as stair climbing. We show that the controller accurately tracks and predicts the user's movements and generates corresponding gaits. Furthermore, based on the modular control architecture, the controller can be extended to support various distinguishable gaits depending on differences in sensory feedback.

## 1. Introduction

Bipedal gait is inherently unstable (Winter, 1995; Milton et al., 2009) and therefore requires constant balancing and support from the lower limbs. This inherent instability can be amplified by changes in nerve, muscle, or bone status. Consequences can range from limited mobility to a complete inability of effective locomotion. Many different supporting techniques have been developed to regain mobility, e.g., crutches, splints, and prostheses, depending on the physical condition. Here, we focus on a knee-ankle-foot-orthosis (KAFO), a device which is attached to the lower limbs and provides mechanical support to its users.

Selecting and fitting such a supportive device for a patient are performed by professional staff. Based on a given patient's condition, the professional staff determines a device providing the support needed to enable or improve the patient's locomotion. This choice has to take into account the patient's remaining abilities, balancing the patient's need for support against the danger of excessive support which might prevent use of the patient's remaining abilities. In this light, it is advantageous to maximize the patients' own contribution to train their remaining abilities pursuing the aim to preserve or regain the patients' mobility. This increase in mobility has, for example, been observed in terms of the range of kinematic parameters and walking speed (Irby et al., 2005, 2007).

From the patient's perspective, other factors contribute to the assessment of the chosen device and thus to the actual use or even abandonment of a device. For example, comfort in daily activities, the ability to fast and easily don/doff the device, and cosmetic properties, i.e., how the device alters the self-perception, or the perception of others (Bernhardt et al., 2006; Robinson et al., 2010; McKee and Rivard, 2011), have a high impact on patient satisfaction. Kaufman et al. (1996) presents several studies where abandonment rates “from 60 % to nearly 100 %” were observed. In Phillips and Zhao (1993), from 60 users of lower extremity braces, 35 abandoned their devices. In the list of top reasons, the authors cite whether the user's “personal opinion [was] considered in the selection process.” Interestingly, it was not important if there were “alternatives to choose from.” A follow-up survey on 250 veterans after 22 months of rehabilitation programs showed that only 16 out of 73 contactable patients were still using their braces. In other words, 78 % had abandoned their devices. In another example, in 31 % of 35 replies, patients expressed that they did not use their brace anymore while “60 % continued to use their wheelchair as their main means of displacement” (Mikelberg and Reid, 1981). Although the studies on device abandonment are from the 1980s and 1990s, they make clear the importance of the patient's opinion concerning the prescribed brace.

Another problem is side effects of orthosis use, which can arise from device limitations or mistrust toward its reliability. Gailey et al. (2008) gives an overview on gait deviations of prosthesis users, including a tendency to favor the intact limb, generating additional stress on the less impaired parts of the body which may induce secondary conditions. There are, for example, (1) degenerative changes (trunk asymmetry, osteoarthritis, and scoliosis), (2) pain (lower back, hip, and/or knee joints), and (3) general deconditioning. These changes can, for example, be observed as asymmetric and slower gaits. Compensatory gait patterns like “increased upper-body lateral sway, ankle plantar flexion of the contralateral foot (vaulting) hip elevation during swing phase (hip hike) or leg circumduction” are listed especially for orthoses with fully extended knees in Yakimovich et al. (2009). Mills et al. (2010) come to the conclusion that “there is a large amount of variability with regard to how patients respond to orthoses.” These studies suggest that the patient's gait and device-perception can be improved by individual fitting. As a device-induced gait change surfaces on long timescales (Irby et al., 2005, 2007), Robinson et al. (2010) are speaking about a “lifetime of adjustments,” an approach for individual fitting has to facilitate continuous adaptation.

In consequence, to achieve patient acceptance in addition to an optimal medical outcome, one has to consider the user's impression of the device and its fitting as well as the specialist's opinion.

When looking at controller techniques, finite state machine based controllers (FSMs) provide a large fraction of state of the art approaches. In a FSM, the supported gaits are represented by a number of states, which define the control output. Given specific conditions, transitions between these states occur. To achieve adjustment in such an approach, parameters defining the control output or transition conditions may be changed. The number of states and transitions is typically left unchanged, with exceptions like Zlatnik et al. (2002), where a rule database is used. As the complexity and variety of the supported behaviors directly translates to the complexity of the graph, a higher number of states and transitions are needed. The process of designing or extending gait support has to ensure that the controller will not get stuck and that transitions support all possible changes in patient behavior.

Approaches to provide complex behaviors, i.e., more gaits, have to deal with increasing complexity. For example, a controller, consisting of three FSMs for walking, standing and sitting movements, is presented in Varol et al. (2010), where additional transitions switch between these individual FSMs. In Sup et al. (2011), a similar approach handles slopes of varying degrees, using three FSMs for level ground, as well as 5 ° and 10 ° inclinations. These approaches try to reduce FSM complexity by a divide-and-conquer approach, but only tackle sub-problems, e.g., either level and slope walking or level walking, standing, and sitting. A similar approach is gait (FSM) switching based on a Gaussion mixture models (Varol et al., 2008). The switching is based on a history of means and standard deviations in the input channels treated with dimension reduction; inputs are sampled with 1, 000 Hz. The behavior was optimized in terms of increasing the history length, resulting in a switching delay of 430 ms in the testing condition.

Other approaches try to provide adaptive control. Speed estimation (Herr et al., 2002) and slope estimation in standing (Lawson et al., 2011) try to achieve control which adapts to gait and environment by the adjustment of control parameters at runtime. These approaches are limited by the flexibility designed into the underlying state machines.

These controllers select between gaits chosen at design time and are partially able to adapt to environmental changes or walking speed. The included FSM controllers represent predefined gait models, only allowing to fit the behavior to the patient with design-time selected parameters. With more supported movements comes higher complexity in terms of an increase in states and possible transitions, which allow more parameters to be selected, still the designed gait model may not cover every individual gait. This problem of fitting the controller to the patient gets worse in case of orthoses, where the patient's conditions are more variable. The variability in patients conditions results in large variability in remaining abilities and, thus, in large variability in individual need for support. These varying conditions are often met with very individual avoidance or compensation strategies, resulting in unique gaits which can conflict with the general gait model.

In consequence, we identified five important problems, which dominate the success of a device: (i) *individualization* according to the patients' neurological status and remaining motor function. As a complication, orthosis patients can show very differentiated medical conditions which have to be compatible with the devices' support. (ii) Typically devices have a *specialized design* supporting a reduced set of movements, which limit the patients' mobility. (iii) The *target group* of orthotic devices is typically limited as a consequence of (i) and (ii). (iv) *An asymmetric gait* due to patients' favoring of healthy limbs leading to gait deviations and secondary conditions. (v) *Device acceptance* is strongly subjective and depends on users' opinions toward the device and their role in the selection process.

These problems have so far not been approached with a common concept. Here, we assume, that they can be addressed best on the controller side with a shift of focus on extensive patient fitting and behavior adaptation. Thus, we propose a personalized and patient centered approach, which *individualizes* via training with patient data. With an user interface, patients directly influence the control output, giving them direct feedback on a possible tuning. The patients' gaits are tracked in terms of gait dynamics, i.e., joint-sensor dynamics. This approach of relying on the sensor dynamics makes the controller independent of the actual mechanical structure of the device as well as, for example, the moments or joint angles the patients can apply and maintain. As controller training leads to a high affinity to the gait dynamics of the trained gait, a modular structure is presented. In this modular structure, the number of supported gaits is limited by the systems' ability to differentiate the gaits by their dynamics. These dynamics are determined by the chosen sensors. As the design is not geared toward a specific set of sensors, the modular controller is not explicitly limited toward specific movements. Thus, given suitable data, which signifies the new gait, the extension with a new gait is a simple, formalized process. With this approach, we want to overcome *design limitations* and extend the *target group*. Furthermore, training of *individual* gait with direct patient feedback in the tuning process may lead to a better fitting and understanding of the controller behavior, hopefully leading to more *symmetric gait* and better *device acceptance*.

Taken together, in this study we present gait dependent damping modulation based on gait phase tracking. Gait phase tracking is based on observed gait samples and therefore implements personalized gait support for single gaits. The damping modulation is implemented as a one dimensional mapping from the gait phase to the desired damping, which can be adapted via a simple user interface. Together, an implementation of (a) gait phase tracking paired with (b) suitable damping modulation constitutes a supported gait. The second contribution lies in the selection of a suitable gait from a set of supported gaits, allowing to adapt the controller's behavior to gait changes. The most suitable gait is selected based on the gait dynamics, which is predicted by internal models for the gait dynamics. Thus, for the second contribution, now three components constitute a supported gait: (a) gait phase tracking, (b) suitable damping modulation, and (c) a model to predict the gait's dynamics for gait identification. Gait selection on three such defined gaits, for walking on flat ground, stair climbing, and descending stairs, have been tested on a healthy subject with an orthosis prototype provided by Otto Bock. This prototype applies damping to knee flexion, providing support to the users' body. Based on the tests with this prototype, we provide performance data on the method's ability to linearly track the gait phase, as well as its ability to fast and reliably select a suitable single-gait controller. As our long term goals of the study, we aim to implement a fully adaptive controller with the patient's user-feedback. The feedback mechanism will not only enable the user to influence the devices behavior, but also provide the means to control changes made by an adaptive controller.

## 2. Materials and methods

**Figure 1:**
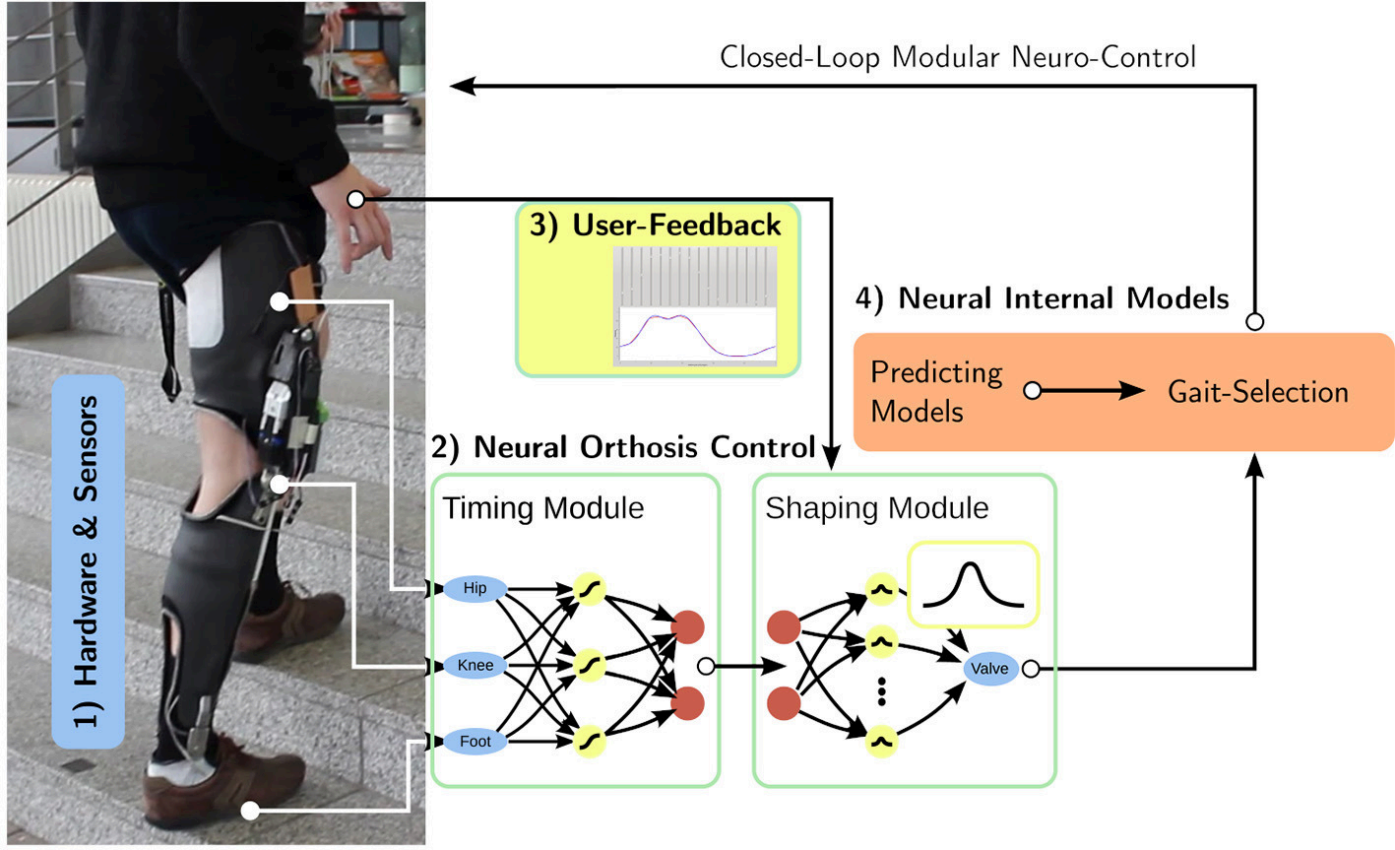
Overview: Based on (1) the hardware platform and its sensors, we develop (2) neural feed-forward gait controllers consisting of a timing and an output shaping module. While the timing unit learns the user's gait from observation, the shaping module is controlled via direct (3) user input, integrating the patient in the control loop. The whole controller consists of several feed-forward controllers which are augmented with predicting gait models. Based on these (4) internal models, the controller selects the correct gait for the current motion.

In the introduction, we outlined the five general problems we see with current control schemes and our approach to them, like individualization, fitting, and behavior adaptation based on patient gait data with the inclusion of user feedback. Here, we outline the implementation based on the concrete control scheme (Figure 1) for gait phase tracking, prediction, and selection.

We present the hardware platform together with the sensors capturing its configuration in section 2.1. In section 2.2, we introduce the single gait controller as the core neural control module. It consists of gait phase tracking, the timing module, and the shaping module which transforms the gait phase into the control output. The single gait controller relies on the user interface to gather user-feedback (in section 2.3), which is only considered here, as it provides the control output as a function of the gait phase. Based on single gait control, section 2.4 presents predicting gait models and the gait-selection module, which will select a single-gait controller in accordance with current motion. As fundamental basis for the analyses, we describe how the segmentation of continuous recordings into steps has been performed in section 2.5. Finally, the experiments underlying this manuscript are presented in section 2.6.

### 2.1. Hardware

The hardware platform used during controller development and for tests with healthy walkers is based on the Otto Bock C-Leg® hydraulic damper attached to the knee joint of a knee-ankle-foot-orthosis. The damper allows the design of a semi-active orthosis, as it actively manipulates damping of knee-flexion with a motor-controlled valve. The interface allows to position the valve in 100 configurations from effectively free motion (open valve) over high damping to blocked motion (closed valve).

**Figure 2:**
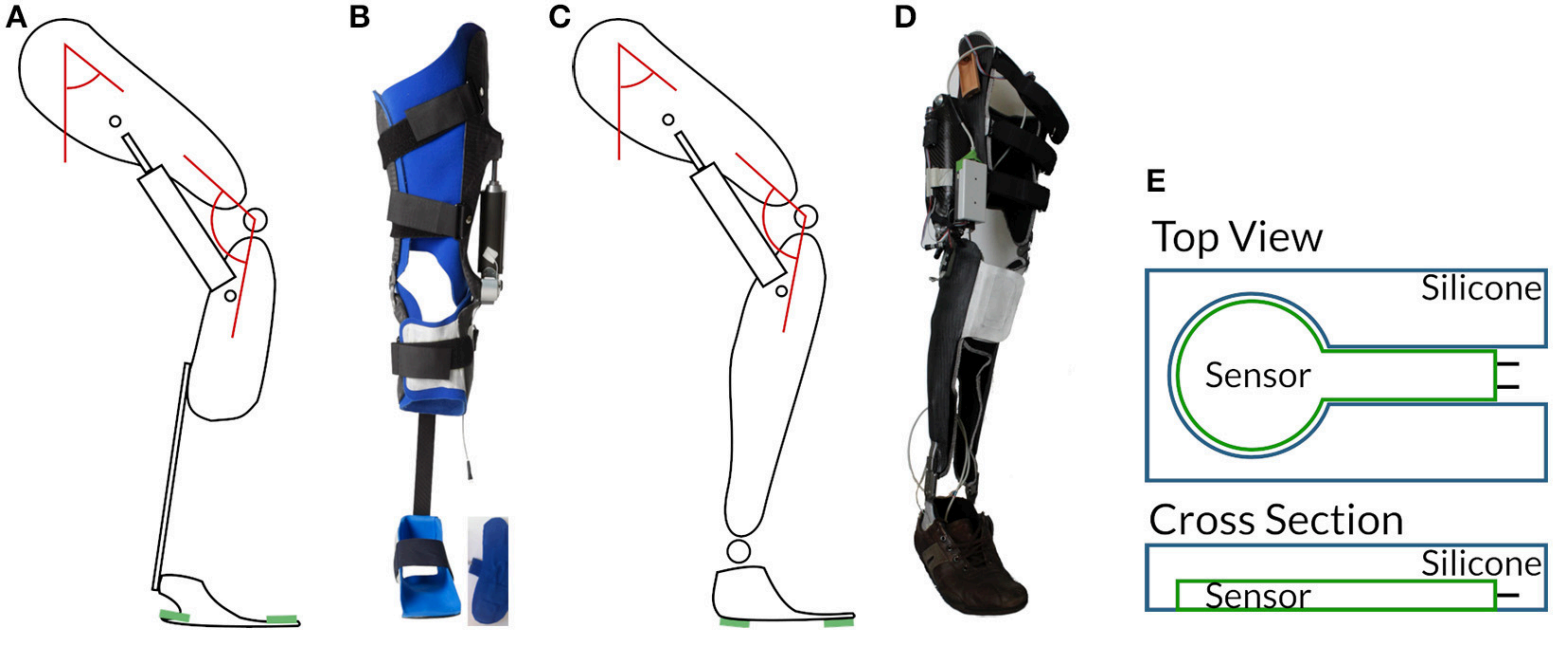
Hardware revisions: In **(A,B)** the first generation with compliant ankle, in **(C,D)** the second with orthopedic ankle joint. The schematics include positions of thigh- and knee-angle sensors indicated by red angles and pressure sensors in green. Force sensing resistors (FSRs) are embedded between the orthosis frame and the shoe. To protect the highly sensitive FSRs against interactions between the orthosis frame and the shoe, we embed them in a silicone plate **(E)**. The applicability to different different hardware layouts shows how versatile the presented controller is.

While the overall design as leg splint with the knee damper system restricts the target group, we aim to have the controller as universally applicable as possible. Therefore, we implemented and tested with two hardware models (Figure 2). One hardware model has a compliant ankle joint (Figures 2A,B). It uses a carbon fiber bar of high stiffness beneath the knee which is directly attached to the foot, thus fixating the ankle. The other one has an orthopedic ankle joint (Figures 2C,D). The orthopedic ankle joint allows either free motion or to constraint the range of motion by blocking it or inserting a spring (comparable to the Otto Bock double action joints). Both models' hardware structure is similar to Otto Bock's C-Brace® system, which is equipped with a C-Leg® hydraulic damper itself. As each device was fitted for a different healthy user, the data wouldn't have been directly comparable. For this reason, we only include data from the device with the orthopedic ankle joint in this study, while pointing out that the controller itself is independent of the actual hardware structure.

A data acquisition interface allows sampling of the embedded sensors at 100 Hz. As sensors, we equipped angle sensors at the thigh and the knee-joint, and force sensing resistors (FSRs) in the soles between orthosis frame and shoe. The latter are very sensitive and show a binary switch characteristic due to their measuring range of ≈ 0.1 − 100.0 N. We therefore embedded them in a silicone layer to reduce noise from interactions between the orthosis frame and the shoe. We localized and fixated all sensors on the device to keep the procedure of device application as simple as possible. Additionally, sensor calibration to achieve full range input signals for the artificial neural networks does not have to be recalculated when reequipping the device.

### 2.2. Neural control for gait tracking

The application of damping to knee-flexion is a one dimensional control problem, where the controller determines a valve position regulating the desired damping. We assume, that the required damping can be determined from the gait phase, i.e., the configuration of the leg as represented by a suitable set of sensors. In practical situations, the controller has to cope with huge variances in space and time. Here, the controller is designed as a feed forward controller to achieve an immediate response to sensory inputs $\vec{s}\left(t\right)$ in two steps. The *timing unit* estimates the phase of the gait φ(*t*) from the sensory reading $\vec{s}\left(t\right)$, while the *shaping unit* determines the damping *c*(*t*) = *c*(φ(*t*)) given the phase (Figure 1). Separating these two steps allows to independently modify the gait tracking and the desired controllers behavior.

For the time discrete control system we write at time step *t*:

(1) $$\begin{array}{ll} \text{timing unit}:\; & {\vec{s}}^{t}\mapsto \varphi^{t},\varphi^{t}\in \left[0,1\right) \end{array}$$

(2) $$\begin{array}{ll} \text{shaping unit}:\; & \varphi^{t}\mapsto c^{t}\left(\varphi^{t}\right),\text{with}c\left(0\right)\equiv c\left(1\right). \end{array}$$

We chose to implement *c* with a radial-basis-function network as universal function approximator, as detailed in section 2.3.

**Figure 3:**
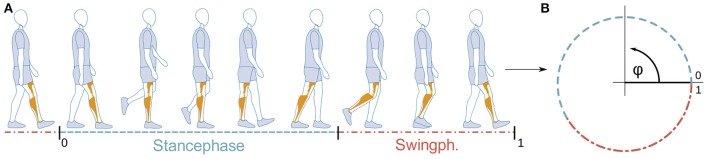
Mapping of gait progress with time **(A)** to the cyclic gait-phase variable φ **(B)**. Restricting φ ∈ [0, 1) allows a direct comparison to classical gait descriptions in the literature. (Illustration **(A)** based on marketing material by Otto Bock).

The gait progress φ is modeled as a cyclic, angular variable (Figure 3), thus capturing the periodicity feature of walking.

The timing unit is implemented using a multi-layer perceptron network with sigmoidal activation function (Nissen, 2003) with four neurons in one hidden layer and two output neurons, representing φ as circular motion in the plane. Thus, the output function is similar to the periodic sensory inputs, which improves learning and accuracy.

$$\begin{array}{l} {\hat{\varphi }}^{t}:{\vec{s}}^{t}\mapsto \left(\begin{array}{c} x_{\varphi }^{t}\\ y_{\varphi }^{t} \end{array}\right)=\left(\begin{array}{c} \cos(2\pi \varphi^{t})\\ \sin(2\pi \varphi^{t}) \end{array}\right). \end{array}$$

In case of noisy sensors, a low-pass filter can be applied on top of the output function $\hat{\varphi }$. With reliable sensors, this step is typically not needed. As it only leads to a small delay, it can be applied anyhow.

The gait phase can then be gained using the transformation

$$\begin{array}{l} \varphi^{t}=\left\{ \begin{array}{l} \frac{1}{4}\text{ for }x_{\varphi }^{t}=0\wedge y_{\varphi }^{t}\geq 0\\ \frac{1}{2\pi }tan^{-1}(y_{\varphi }^{t}/x_{\varphi }^{t})\text{ for }x_{\varphi }^{t}\neq 0\;,\varphi \in [0,1).\\ \frac{3}{4}\text{ for }x_{\varphi }^{t}=0\wedge y_{\varphi }^{t}<0 \end{array}\right.\; \end{array}$$

To facilitate network training, sensor calibration is used to map all values to the range [−1, 1]. The calibration procedure uses: vertical thigh (0), to 90 ° flexion (1). The knee angle is mapped from straight (−1) to 90 ° flexion (1). For the force sensors, thresholds are chosen such that ground contact maps to ≤ 0 and a free foot to 1.

Training data is then segmented into steps (section 2.5) using the ground contact signal. The step duration *l*_*j*_ is determined as the number of samples in the jth step. Then, the desired gait phase φ_*i*_ and network output *o*_*i*_ for each sample *i* are given as

(3) $$\begin{array}{ll} \varphi_{i} & =\frac{i}{l_{j}}, \end{array}$$

(4) $${\vec{o}}_{i}=\left(\begin{array}{c} o_{1}\\ o_{2} \end{array}\right)=\left(\begin{array}{c} -sin(2\pi \varphi_{i})\\ -cos(2\pi \varphi_{i}) \end{array}\right).$$

We chose the sensors with the aim to capture motion in terms of sensor dynamics, instead of relying on defined events. The *timing module* frees the sensory inputs from time dependencies and thus provides a device, gait, and patient independent description of gait progress. The second part, the *shaping module*, generates the control output. It is augmented by a user interface for direct user-feedback.

### 2.3. User defined output modulation

The damping function is tailored to the need of the individual user by incorporating user feedback in the shaping of the damping function *c*(φ).

**Figure 4:**
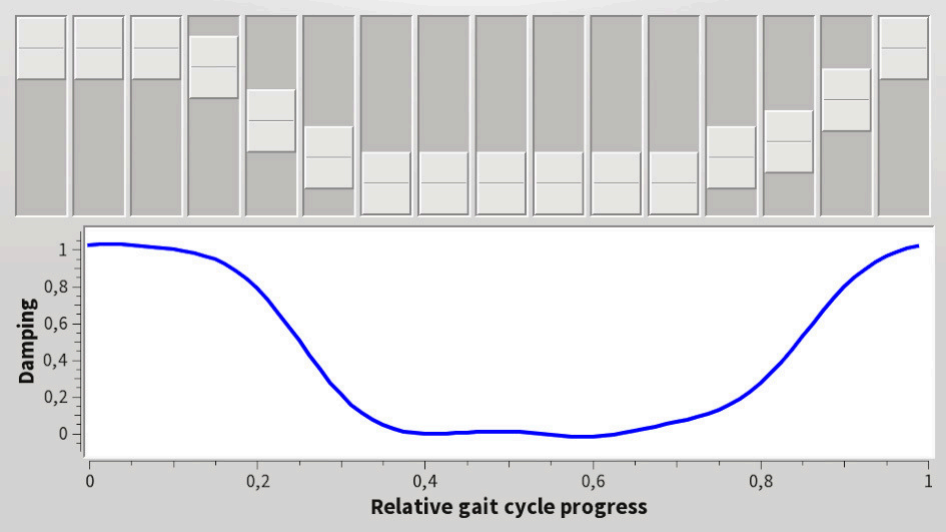
User interface to tune the control output, i.e., the knee damping. The horizontal axis shows the gait phase φ, the vertical the desired damping *c*. Changing a slider, directly modifies the desired damping at the corresponding gait phase. The system updates its behavior immediately to changed user input. This enables users to tune the device's behavior to their expectations and needs.

The user interface (Figure 4) provides the samples to fit the Radial-Basis-Function network, which provides universal function approximation (Park and Sandberg, 1991; Buhmann, 2003). The sliders represent the applied damping *c*(φ) by values on a grid of supporting points, with the lowest position corresponding to no damping, the highest position to maximum damping. The Radial-Basis-Function network is updated immediately and thus users can immediately experience the effect of their changes. The choice of the Gaussian kernels' widths allows to choose the amount of smoothing of user-input applied during network-training.

We used a network of *n* = 10 equidistant nodes in the interval [0, 1). The Gaussian transfer function had a half-width of $\sigma =\sqrt{\frac{n}{2}}$.

### 2.4. Gait recognition

The neural orthosis controller described in section 2.2 is gait specific: its timing and shaping units were designed to estimate the phase of a specific gait and generate the damping appropriate for that gait. To support different gaits, we propose to train different controllers for different gaits, and to activate the proper controller based on model based gait recognition. As long as the gaits can be differentiated with the available sensory information (Figure 1), the number of supported gaits is not directly limited. The controller can be extended by providing single-gait controller modules together with internal models to identify the corresponding gait (Braun et al., 2014).

Gait recognition is based on the prediction of sensory input: each gait is associated with a predictor P that predicts the sensory input ${\vec{s}}^{t+1}$ for the next time step based on a subset of the sensory history $H_{N}=\left\{{\vec{s}}^{t},{\vec{s}}^{t-1},\ldots ,{\vec{s}}^{t-N+1}\right\}$, where N is the history length. A comparison of the prediction of the next time step's sensory reading ${\vec{p}}^{t+1}$ to the actual sensory reading ${\vec{s}}^{t+1}$ in the next time step defines a prediction error. The prediction error allows the decision unit to determine the best fitting gait model by choosing the model with the smallest error within predefined acceptable bounds of error.

To estimate reasonable history lengths N, we assume a minimal step duration *T*_*step*_ of

$$T_{step}\underset{˜}{≿}1\;s,$$

for complete steps with the orthosis. Further, we assume a stance to swing duration ratio of ≈60:40 and that gait changes can occur at any time^1^. When the gait changes, the history contains two gaits and will naturally lead to diverging predictions of models trained on a single gait. While this prediction error is critical to achieve a fast invalidation of the old gait, we want the history to contain only one gait swiftly afterwards. To estimate a reasonable scale of the new gait's duration in the transition step, we go for a fraction of 50 % of the swing phase as the smaller gait phase. This translates to around 20 % of the step length. Given the hardware specific sampling frequency of 100 Hz, we determine the maximum number of samples in the history N_History_ to

$$\begin{array}{l} N_{\text{History}}≾˜20\;samples=\frac{1}{5}\;s. \end{array}$$

When the history length is chosen larger, a gait change could stay longer in the history than the above requested 20 % of the step length.

The history length is a trade-off between the prediction's accuracy and the supported frequency of gait switches. The prediction accuracy should increase with a longer history, which can cover more details leading to better discrimination. In contrast, the frequency of supported gait switches will decrease, because data from different gaits in the history will lead to lower quality predictions while conflicting gaits stay in the history. The choice of $T_{H}=\frac{1}{5}\;s$ allows several gait switches per step with quite accurate results, as shown below.

#### 2.4.1. Predicting gait models

**Figure 5:**
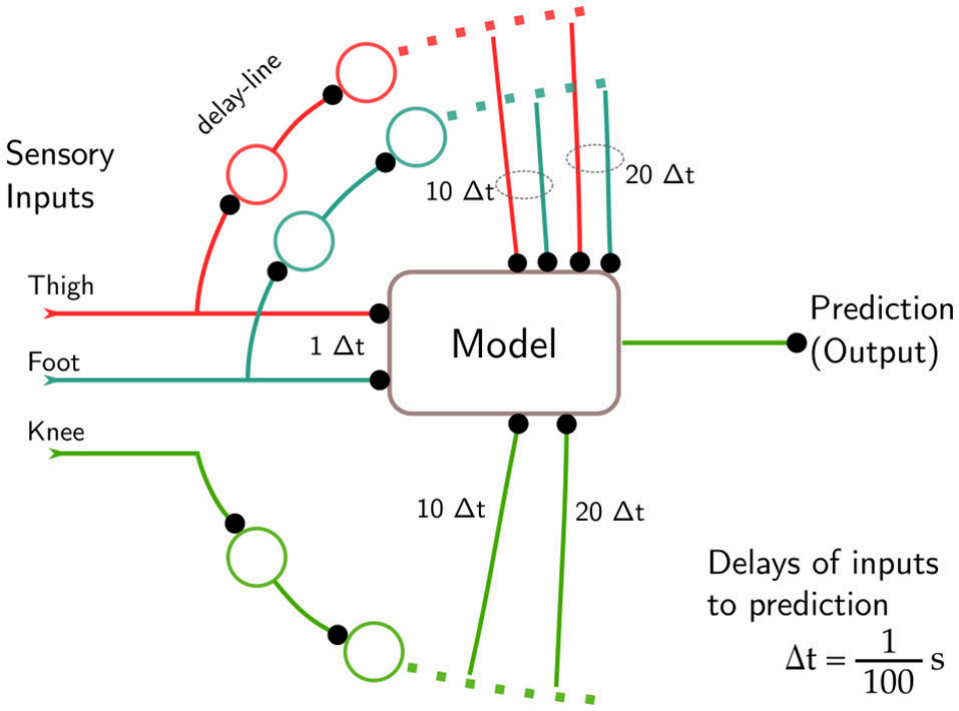
Gait detection tries to minimize the prediction error for sensory inputs ${\vec{s}}^{t}$ by choosing an appropriate model. Delay lines provide a history H of sensory inputs, which is fed into a feed-forward model predicting a single value in the next time step, here the knee. The delay relative to the predicted signal is given as a multiple of the sampling interval 1100 s. For the predicted channel, the current input is not fed in, as experiments showed a high probability of the network to choose it as the best prediction.

The predicting gait models were implemented like the timing unit of the feed-forward gait controller above. Based on a history of sensor data, a single channel was predicted by the predicting model. To this end, a multi-layer perceptron network with 3 neurons in the hidden layer and one output neuron was trained on recorded gait samples to predict the sensory inputs using a history. The history was implemented as a delay line (Figure 5).

First experiments showed that the backpropagation learning algorithm tended to exploit the last sensor reading as a good prediction of the next time step. Ignoring most of the history, this was equivalent to the approximation with a constant value, predicting the next time step's state almost only on the preceding one as the error of the approximation was in many cases of the order of magnitude of the signal change, considering step to step fluctuations and sensor noise. This prediction on only one time step was independent of the trained mode. Thus, this approach had no predictive power which related to the actual gait's dynamics.

As a consequence, only a subset of the history is actually used for prediction. Especially for the predicted channel, the current sensory reading is omitted and only older values are used. Effectively, we have coarsened the history to a grid with a width of 10Δ*t* (Figure 5), to exclude simple models. Besides solving the problem of forwarding of the last reading, this sparse selection reduces the computational complexity of the models greatly.

Therefore, to predict channel i ${\vec{p}}_{i}^{t+1}$, we use sensory readings of all other channels for *t*, *t* − 9, and *t* − 19, but only the readings for *t* − 9 and *t* − 19 of the predicted channel (Figure 5).

#### 2.4.2. Prediction-based gait selection

The part of the controller, which selects the current gait based on the model's predictions, will be called the decision unit, in accordance to previous naming conventions. The selection process chooses the gait model which minimizes the prediction error ${\vec{e}}_{j}^{t}=|{\vec{p}}_{j}^{t}-{\vec{s}}_{2}^{t}|$ for the gait *j* with the sensory inputs ${\vec{s}}_{2}^{t}$. The subscript 2 indicating that the ground contact signal has not been used for the calculation of the prediction error. The absolute value was chosen to increase sensitivity to the amplitude of the prediction error, preventing the low pass filter (below) to average out fluctuations.

These prediction errors often occur in relatively short intervals of the step. Thus, we apply post-processing in form of a low pass filter to prolong the time span that the final fitness-measure is usable.

$$\begin{array}{l} {\tilde{e}}_{i,j}^{t}=(1-\beta ){\tilde{e}}_{i,j}^{t-1}+\beta e_{i,j}^{t},\beta =0.9\text{, }i𠈈\{\text{knee},\text{hip}\}\text{.} \end{array}$$

**Figure 6:**
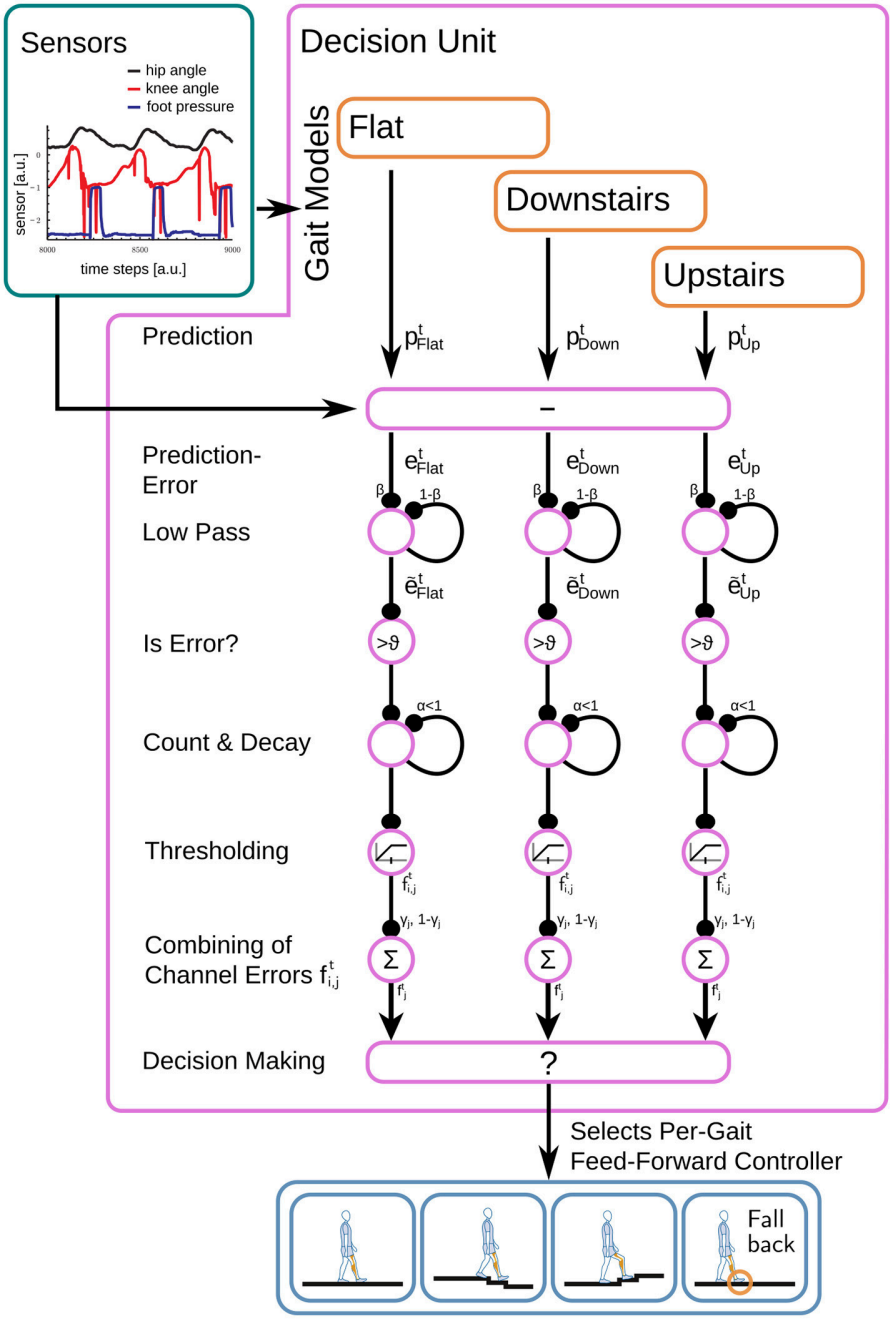
Based on the gait-specific predictions for the next sensory inputs ${\vec{p}}_{j}^{t}$ and the actual sensory reading, the decision unit calculates the prediction error ${\vec{e}}_{j}^{t}$ and applies a low-pass filter before an unfitness measure $f_{j}^{t}$ is calculated for each gait. At the end, from gaits with $f_{j}^{t}$ in an acceptable range, the best fitting one is selected.

Model- (*j*) and channel (*i*) -specific thresholds θ_*i, j*_ suppress prediction noise in the low pass filtered errors ${ẽ}_{i,j}^{t}$. These thresholds are chosen for each gait *j* individually based on the error signal ${ẽ}_{i,j}^{t}$ for matching gait samples. Remaining prediction errors are counted if they are greater than this threshold θ_*i, j*_ (Figure 6) for each predicted channel *i* ∈ {knee, hip}.

$$f_{i}^{t}=\alpha \cdot \{\begin{array}{cc} f_{i}^{t-1}, & \text{if }{\tilde{e}}_{i}^{t}<\theta_{i}\\ \max\left(f_{i}^{t-1}+1,2\right), & \text{else} \end{array}\;,\;\alpha \in \left\{\mathbb{R}|0<\alpha <1\right\}.$$

This count $f_{i}^{t}$ is limited to the range [0, 2] and decays with factor α = 0.99. The factor α and the maximum value 2 are chosen such that the value is significant on timescales of steps.

These $f_{i}^{t}$ measure the unfitness of the model's predictions per channel and are merged with a gait specific weight γ_*j*_ to reflect the importance of the individual channels,

$$\begin{array}{l} f_{j}^{t}=\gamma_{j}\;f_{thigh}^{t}+\left(1-\gamma_{j}\right)f_{knee}^{t}. \end{array}$$

Finally, all gaits with $f_{j}^{t}>1.1$ are discarded and the gait with the lowest $f_{j}^{t}$, i.e., smallest unfitness, is selected from the remaining gaits. Its feed-forward controller operates the current time step.

#### 2.4.3. Training of prediciting models and selection

Training of predicting models is analogous to the feed-forward controller's timing unit and can use the same recordings. The recordings should reflect the variance in the user's gait and should not be too regular. Then, the perceptron is trained using a backpropagation algorithm.

To improve the performance of the internal models, each model scales the sensory inputs such that typical signals lie in the range (−1, 1). Of course, in addition to optimal working conditions, such a scaling will help to differentiate huge changes in amplitude which might be connected to different gaits. In a converse argument, it supports bad predictions for gaits with too low or too high amplitudes in comparison to a model's training data set.

### 2.5. Step segmentation

Segmentation of gait data by step boundaries is needed to create training data as well as for the analysis of the tracking unit's and gait recognition performance. As typical in the literature, the heel-strike marks beginning and end of a step (Figure 3), which we determine by flanks of the pressure onset at the heel FSR. Due to the high sensitivity of the sensors and interaction with the orthosis frame and foot, only onsets can be detected and we have to apply filters to compensate varying amplitudes and fluctuations.

The sensory data is assumed to be in the range [−1, 1] with 1 no pressure and −1 high pressure. To improve robustness, we use a hysteresis to detect state changes, changing to ground contact when the sensor goes below 0, and to free heel when >0.8. Heel-strike detection is implemented with a finite state machine as:

1. Based on the first sample, the state is initialized to *ground contact* or *free heel*.
2. For each sample
  - If *ground contact* and the current sample is *above threshold for free heel*, then change the state to *free heel*.
  - If *free heel* and the current sample is *below threshold for ground contact*, then change the state to *ground contact*.
3. Collect the sample numbers of all touch down events (state changes to ground contact) in a list.
4. As the detection reacts to the steepest part of the flank in the FSR signal, we move the touch down event to the preceding sample which is closest to an FSR reading of 0.8 to determine the onset of heel-strike.

This list of events now describes the heel-strikes in the given recording.

### 2.6. Experiments

#### 2.6.1. Gait phase tracking

For single gait support, the following statements hold. (i) We make no assumptions about when and what kind of support the user needs. (ii) The damping function *c* is smooth (due to the representation as an RBF function). And (iii) the applied damping at knee-flexion is a direct function of the gait phase and thus of the sensory input $c_{t}=c_{t}\left(\varphi_{t}\left({\vec{s}}_{t}\right)\right)$. Thus, the applied damping *c* only changes when the gait phase φ changes and, in consequence, the controller's ability to apply the desired damping at any gait phase solely depends on the properties of the gait phase φ. Thus, the quality of the gait phase φ determines the quality of the control output.

Ideally, the gait phase φ produces a linear mapping for constant motion velocity, as it guarantees the same detail of control for all phases of gait, i.e., control accuracy does not depend on the gait phase. Thus, we investigate the linearity of the gait progress representation and the timing of the heel strike after training. We compare to the ideal gait phase φ′ according to section 2.5, which can only be derived after the heel-strike and therefore has to be acquired for offline processing. To evaluate steps of different duration, we will resample and interpolate each step to 200 samples, leading to an ideal slope of $\Delta \varphi^{\prime }=\frac{1}{200}$. The deviation of the controller's gait phase to these will be investigated in terms of linearity, monotony, and smoothness.

Furthermore, the timing of the gait phase has to match the timing of the step to provide a useful representation for users, such that the tuning of the control output with the user interface (Figure 4) can be done intuitively.

#### 2.6.2. Gait selection

Due to the controller providing body support at the knee level, it assists in the stance phase, while in the swing phase, all single-gait controllers provide free knee swinging. We therefore argue, that the most important aspect for secure and seamless operation is timely gait switching to prepare for the heel-strike. Thus, to evaluate the accuracy of gait recognition, we check that the controller not only classifies the step correctly but also achieves a correct result prior to heel strike. To quantify correctness and timing, we analyze a walking sequence where a healthy user annotates the intended gait, for example flat walking, stair climbing, and descending stairs. The inclusion of descending stairs requires that we have to deactivate the damping unit for security reasons.

**Figure 7:**
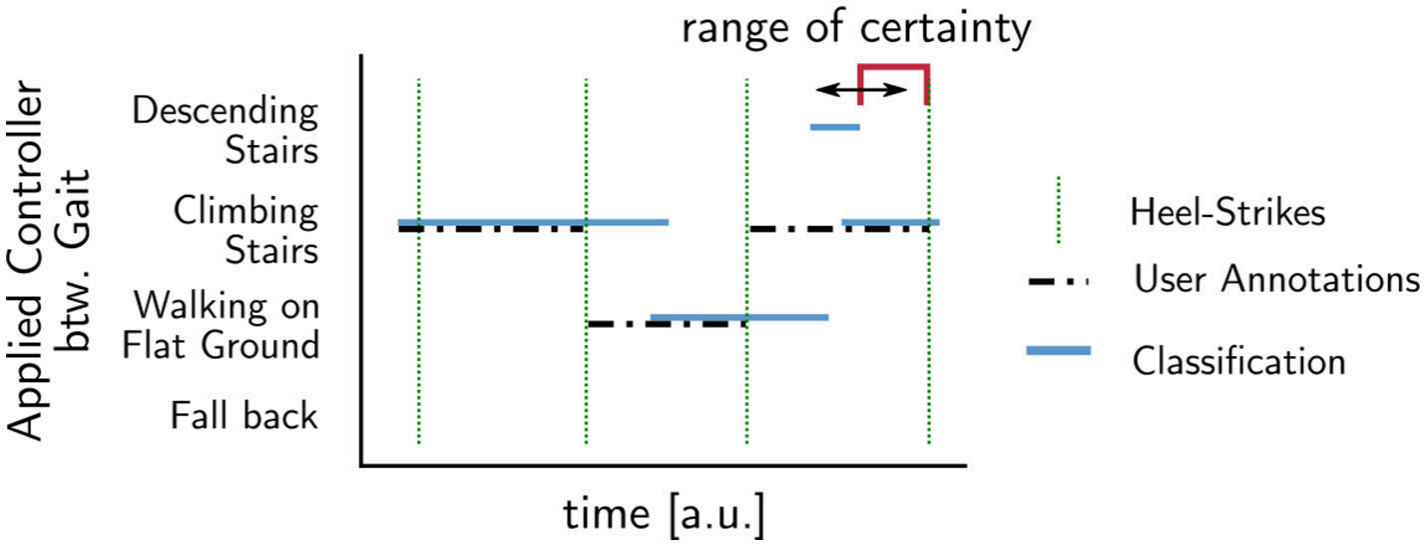
Determination of the time a correct classification result was available before heel-strike: The *range of certainty*, i.e., the fraction of swing phase where user annotations (dotted black) and the system's prediction match. It describes the duration for which the prediction produces a correct result and describes the predictive power of the model for a specific step.

Then, we analyze step by step and measure the time ahead of the heel-strike that the decision unit recognized the step's final gait. Figure 7 shows how the user's annotations are compared to the controllers' classification: For each step, we compare the controllers' classification against the last valid user annotation. If both match, we measure the duration the correct classification was known and set this duration in relation to the duration of the swing phase to allow the comparison independent of the actual step length. We call this fraction the *range of certainty*. A range of certainty of zero means that the correct gait was not known prior to heel-strike. For a range of certainty of one, the controller was certain of the used gait for the whole swing phase.

Thus, the range of certainty allows to asses if the controller is able to apply the correct gait model during swing phase, where all single-gait models will provide free knee motion. We then analyze the average success rate and range of certainty for all tested gaits, to determine if the presented controller in combination with the sampling frequency of the data acquisition board can react to gait changes. Then, we quantify the controller's ability to differentiate the tested gaits against each other with the selected set of sensors. We conclude with the investigation of gait changes for steps showing conflicts between the user's annotation and the controller's classification.

To access the orthosis controllers accuracy, we take a reaction time into account. At a sampling rate of 100 Hz and step lengths in the experiment between 1.3 and 1.8 s, a *range of certainty* of 3 % guarantees that the orthosis controllers' classification is in time for heel-strike.

## 3. Results

### 3.1. Gait phase tracking

The experiments conducted aim to show that a trained gait model is able to track gait progress better than a model trained for other gaits. Control quality depends on the smoothness and monotony of the tracked gait phase φ, which we quantify in terms of linearity and the distribution of increments, e.g., discrete slopes. Furthermore, the accuracy of the tracked heel-strike is used as a measure for correct timing.

In a first run, the single gait controllers were trained on runs with 49 steps on even ground and 59 steps on stairs. In a second run, we record the gait phase φ of these two controllers for later comparison to the ideal gait phase φ′. We analyzed 30 steps on even ground and 38 steps climbing stairs of a healthy subject wearing the orthosis. Steps at gait changes were manually removed, due to issues discussed later.

**Figure 8:**
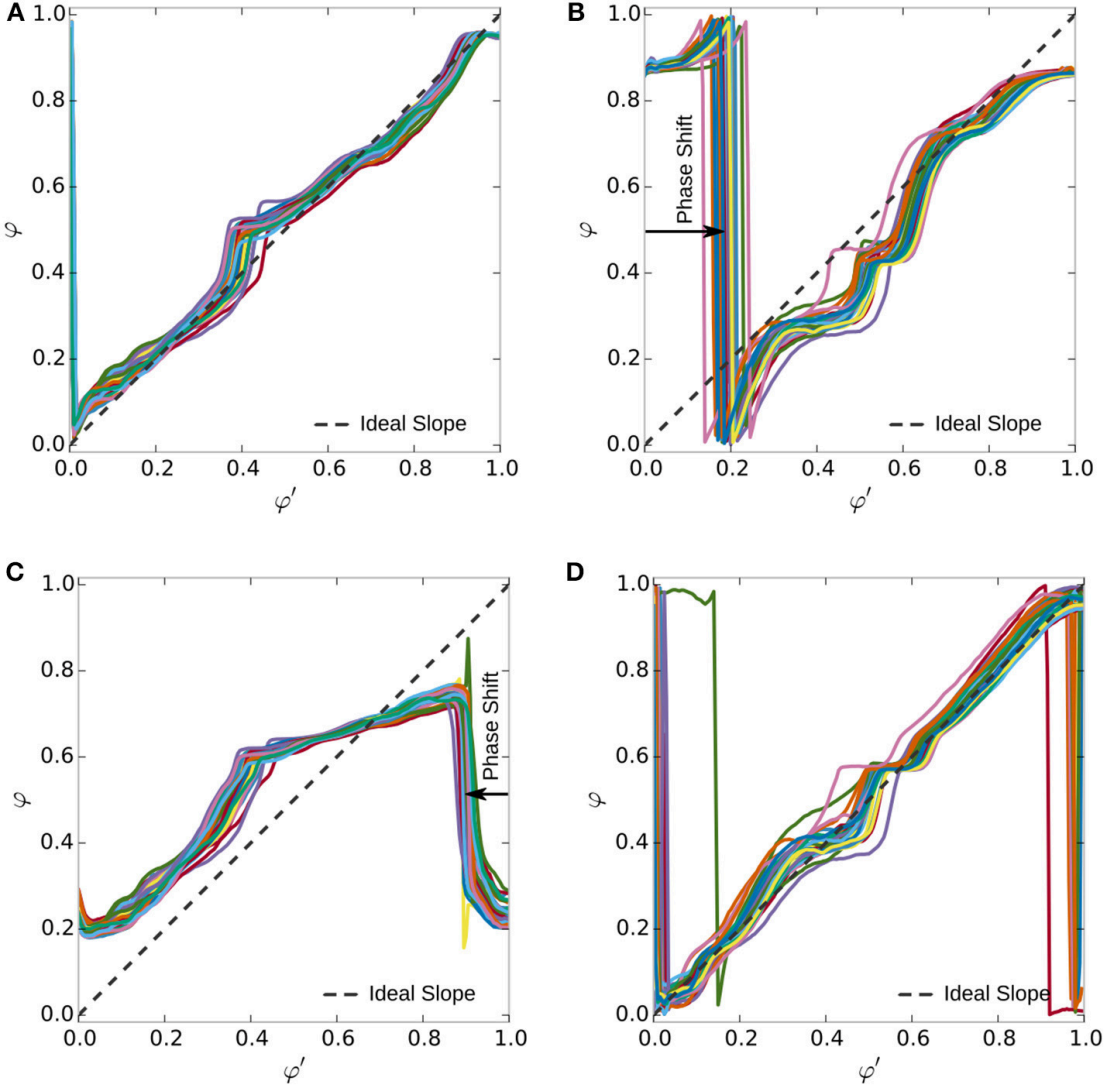
Smoothness of gait phase representation. Colored lines indicate 25 steps on flat ground and 8 on stairs, without gait transitions. Compared are models for walking on flat ground and stair climbing on their native and the opposite terrain. The native models (**A** *r*^2^ = 0.88, **D** *r*^2^ = 0.72) produce smoother gait phase output in comparison to the unfitting models (**B** *r*^2^ = 0.33, **C** *r*^2^ = 0.06). The latter expose phase shifts and strong deviations the ideal gait phase φ′ indicated by the dashed line. The coefficient of determination (*r*^2^) supports the notion that the native models are generally following the ideal linear relation. The lower number of steps in **(D)** increases the influence of the step segmentation, degrading *r*^2^.

In Figure 8, the controller-derived gait phases φ_Flat_ and φ_Stair_ are plotted against the ideal, offline computed gait phase φ′. In the case where the model matches the user's gait (Figures 8A,D), the ideal gait phase is approximated well. In the mixed cases (Figures 8B,C), where the model does not match the gait, the controller's heel strike has a phase shift against the real event. In addition, the flat ground model on stairs (Figure 8B) shows 4 steps with almost constant intervals between the steps. The stair climbing model on flat ground (Figure 8C) fails to reproduce the gait phase completely; it only oscillates between 0.2 and 0.8. The *r*^2^ values in Table 1 support that the native model is close to linear and significantly better than the non-native model.

**Table 1:** Linearity of the graphs in Figure 8 according to the *r*^2^ values for a linear regression.

		**Environment**							
		**Flat ground**				**Stairs**			
		***r*^2**	**Accuracy [°]**	**Std. dev**.	**Skew**	***r*^2**	**Accuracy [°]**	**Std. dev**.	**Skew**
Model	Flat	0.88	1.8	0.004	3.94	0.33	34.2 ± 3.7	0.033	1.17
	Stair climbing	0.06	−18.0 ± 1.2	0.017	−10.45	0.72	2.5 ± 5.5	0.005	2.02

**Figure 9:**
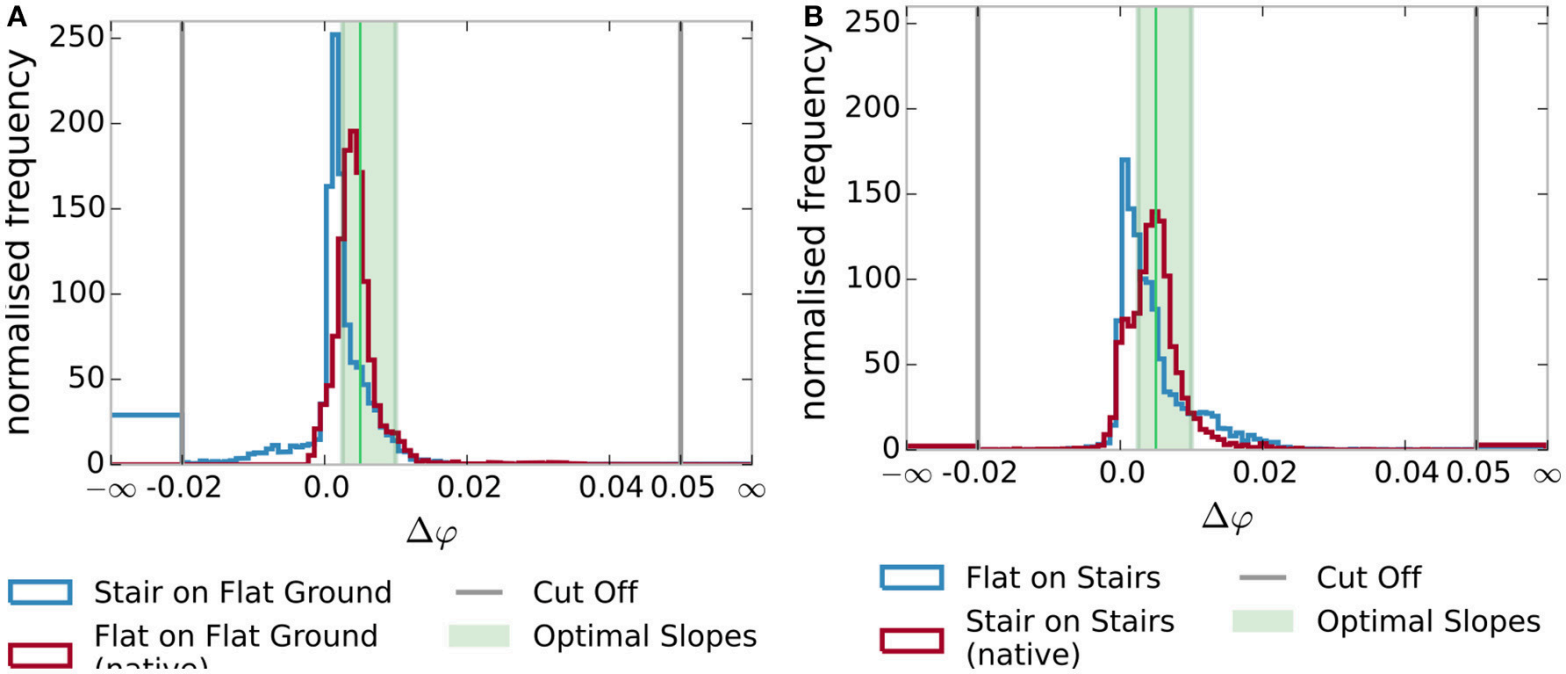
Distribution of gait phase increment sizes Δφ. The native models (in red) have a distribution of increments centered near the ideal linear slope. The non-native models (in blue) show a stronger deviation from the ideal slope. The standard deviation and skewness are noted in Table 1. **(A)** Increments on flat ground. **(B)** Increments while stair climbing.

*Timing accuracy of heel strike based on the heel-strikes' phase shifts as shown in Figure 8. The standard deviation and skewness relate to Figure 9. For flat ground based on 30 steps and while stair climbing (38 steps for the flat model and 31 steps for the stair climbing model)*.

The accuracy in timing of the heel-strike is accurate only for the trained gait, as the phase shift in Figure 8 and Table 1 shows. Considering the sampling frequency of 100 Hz and an average duration of 150–200 samples, the gait phase should progress by $\frac{360\;{}^{\circ}}{200}\text{–}\frac{360\;{}^{\circ}}{150}=1.8\;{}^{\circ}\text{–}2.4\;{}^{\circ}$ per sample. This value matches with the average precision shown in Table 1, which is determined by averaging the phase shift indicated in Figure 8.

The distribution of increments Δφ in Figure 9 supports these observations. When considering the variation of increments around $\Delta \varphi^{\prime }=\frac{1}{200}$, we considered the interval $\left[\frac{1}{2}\Delta \varphi^{\prime },2\Delta \varphi^{\prime }\right]$. Using this interval, we allow a variation of up to a factor of two in each direction, but do not count extreme or negative increments, as the standard deviation might have. For level walking (Figure 9A), the fitting model has 69 % of increments in this interval, while the model for stair climbing only has a fraction of 31 % inside this interval. In the case of steps on stairs (Figure 9B), 65 % of the increments are inside for the fitting model and only 40 % for the flat walking model. The histogram for the native models (in red) has its maximum around the optimal slope with lower standard deviation (Table 1). For the mixed cases (in blue), the optimal slope is not in the center of the distributions but has a maximum around zero and larger standard deviations. Furthermore, we see the presence of significant negative changes for the stair climbing model on flat ground and an increase in larger values in the case of the flat ground model on stairs, i.e., less monotony and smoothness.

**Figure 10:**
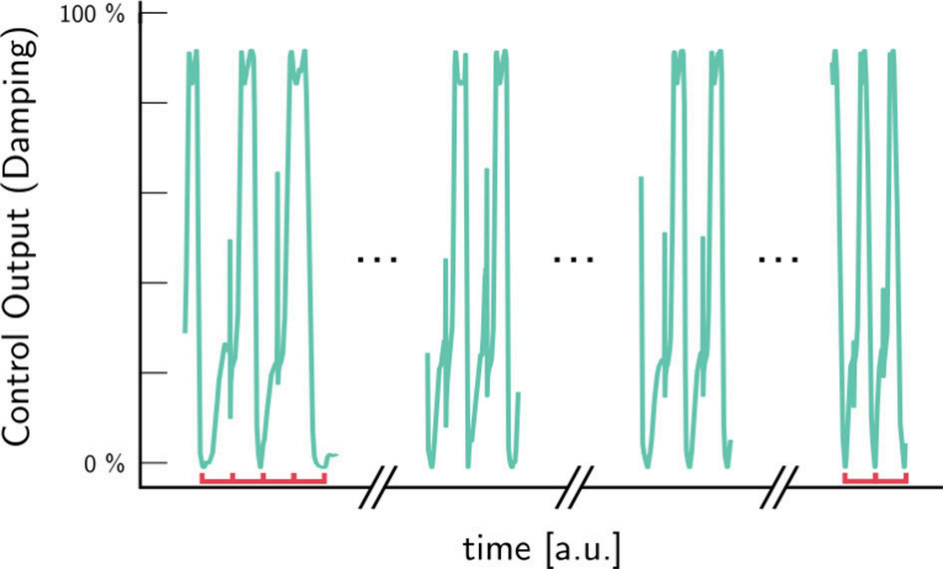
Automatic adaptation to different step-lengths. The abstract gait progress φ removes any time dependency from the input. Step duration and length are transparently handled. The red bars indicate unit lengths: the steps to the right are twice as fast as the ones to the left.

The ability to apply a damping pattern to steps of varying length is shown in Figure 10. As the abstract gait progress φ removes any time dependency from the input, changes in step duration and length are transparently handled. The red bars in Figure 10 indicate unit lengths: the steps to the right are twice as fast as the ones to the left.

### 3.2. Gait selection

In this section, we test the hypothesis that a set of feed-forward single-gait controllers can be combined into to a multi-gait controller that enables the correct feed-forward controller to support a wide range of motions. Therefore, to evaluate the accuracy of gait recognition, we have to show that the gait recognition provides a correct result and that this result is available in time for the controller to react to gait changes.

The experiments include walking on flat ground, stair climbing, and descending stairs performed by a healthy subject. Prior to use, the gait models were trained with 146 steps for walking on flat ground, 35 steps for stair climbing, and 32 steps for descending stairs. The difference in training set sizes is due to every stair run including steps of flat ground and the gaits on stairs being comparatively exhausting. Three independent recordings with 215 steps were used in the evaluation. These include gait transitions between 81 steps on flat ground, 64 steps mixing flat ground and stair climbing and 70 steps mixing flat ground and descending stairs. As the staircase used in the experiment is comprised of sequences of 5 stairs, each of the mixed runs includes the high number of 36 transition steps.

**Figure 11:**
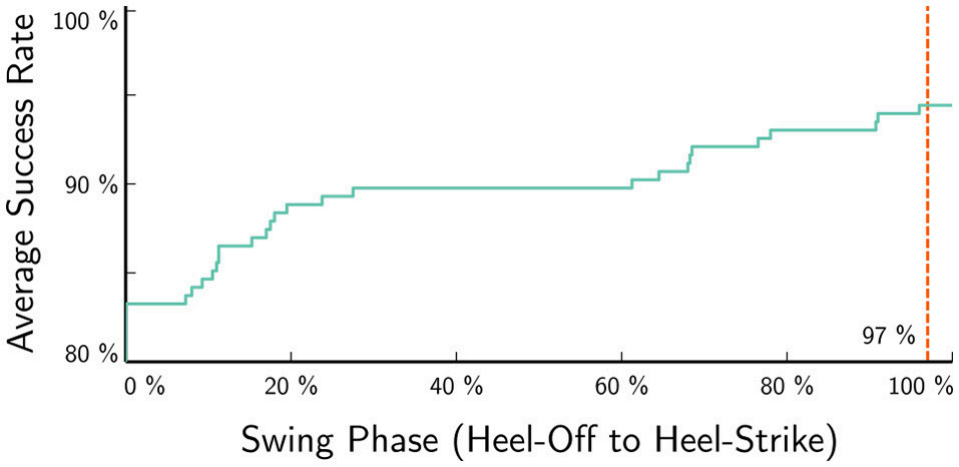
The average success rate increases during swing phase, indicating that the classification matches the user annotation better the smaller the timescale of the prediction is. A high onset of more than 84 % indicates step sequences of unaltered gait. The red line to the right indicates the time, where the controller can still use gait information before heel-strike at ≈97 % swing phase (3 % *range of certainty* Figure 7), e.g., to successfully apply pre-damping.

The development of gait certainty over the swing phase (Figure 11) shows that the gait for 83 % of steps was known at toe-off. The fraction of correct classification now increases toward above 94 % at heel-strike. This high accuracy is indicative of the fact, that most steps stem from step-sequences with the same gait. Furthermore, it indicates that many gait changes occur during swing phase.

**Figure 12:**
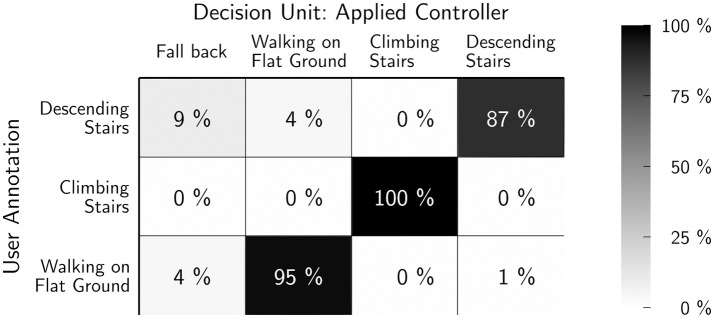
Classification accuracy for a *range of certainty* of at least 3 %. The rows show the user annotation, the columns show the system's output. Note that the latter has the additional category “unkown/fall back” which is selected, when all predicting models generate high errors. Stair climbing has a high accuracy due to its unique phase relation, which leads to a 100 % success rate. Flat walking and stair descent have a higher overlap. Of 215 steps, only 2 transition steps were wrongly categorized (Figures 13, 14).

The final classification accuracy, with ranges of certainty of at least 3 %, are plotted in Figure 12 as confusion matrix between the user annotations in the rows and the controllers' classification in the columns. Note that the additional column *unknown gait* in the controllers' classifications, which counts cases, where the prediction errors are unacceptably high for all gait models. In these cases, the application of a fall back controller allows safe operation, for example, knee locking on ground contact, although it is most likely less comfortable. In general, the confusion matrix shows high classification rates between 87 % for descending stairs, 95 % for walking on flat ground, and 100 % for stair climbing. Furthermore, we see a number of steps, where the gait recognition was unable to differentiate or even mixing up walking on flat ground and descending stairs. The wrong classified steps are one transition step each for descending stairs and walking on flat ground.

**Figure 13:**
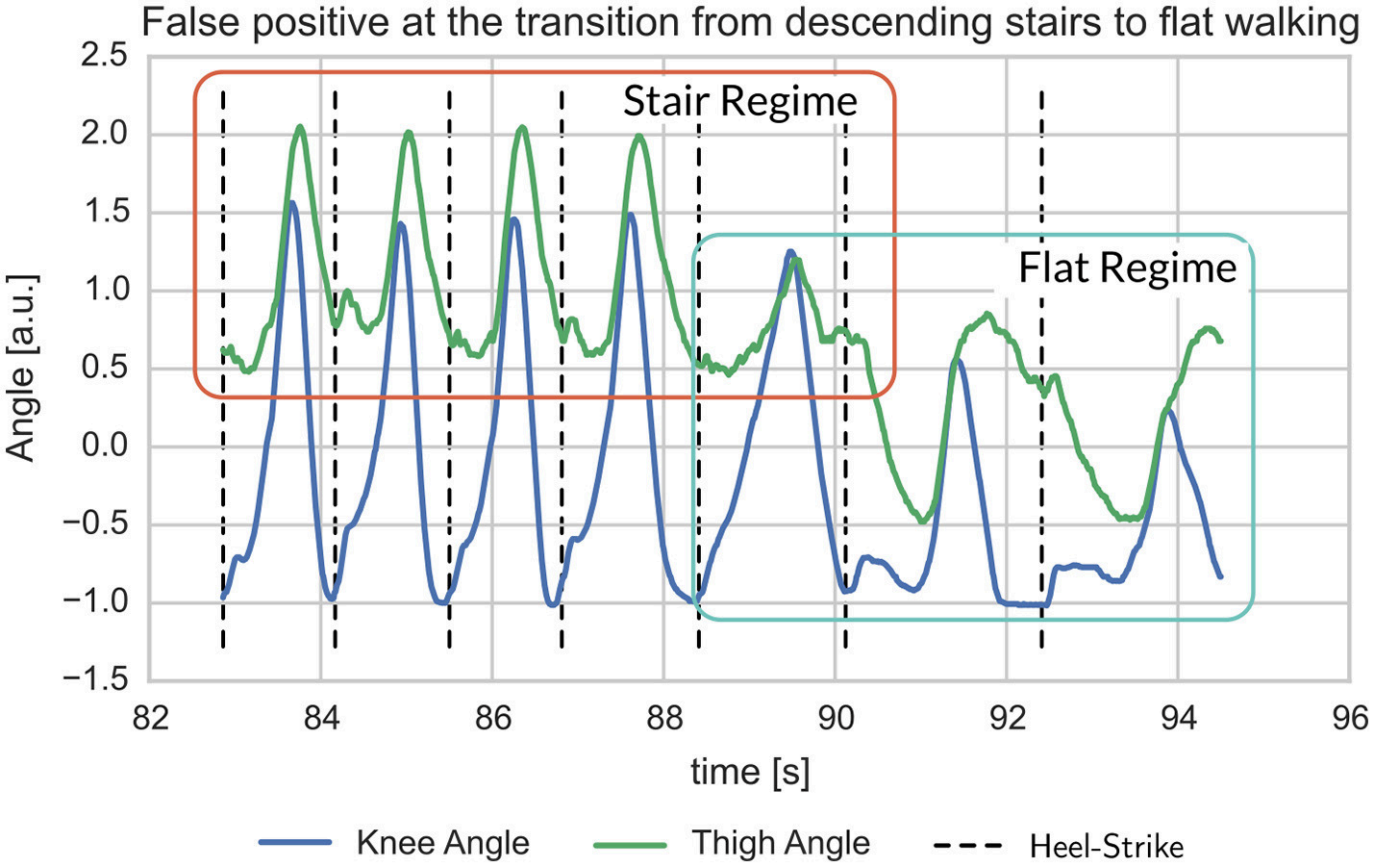
Example for mismatch between user-label and gait detection at the transition from stair descent to flat walking. The transition step clearly deviates from earlier and following steps in that it shows mixed characteristics (Figure 14).

**Figure 14:**
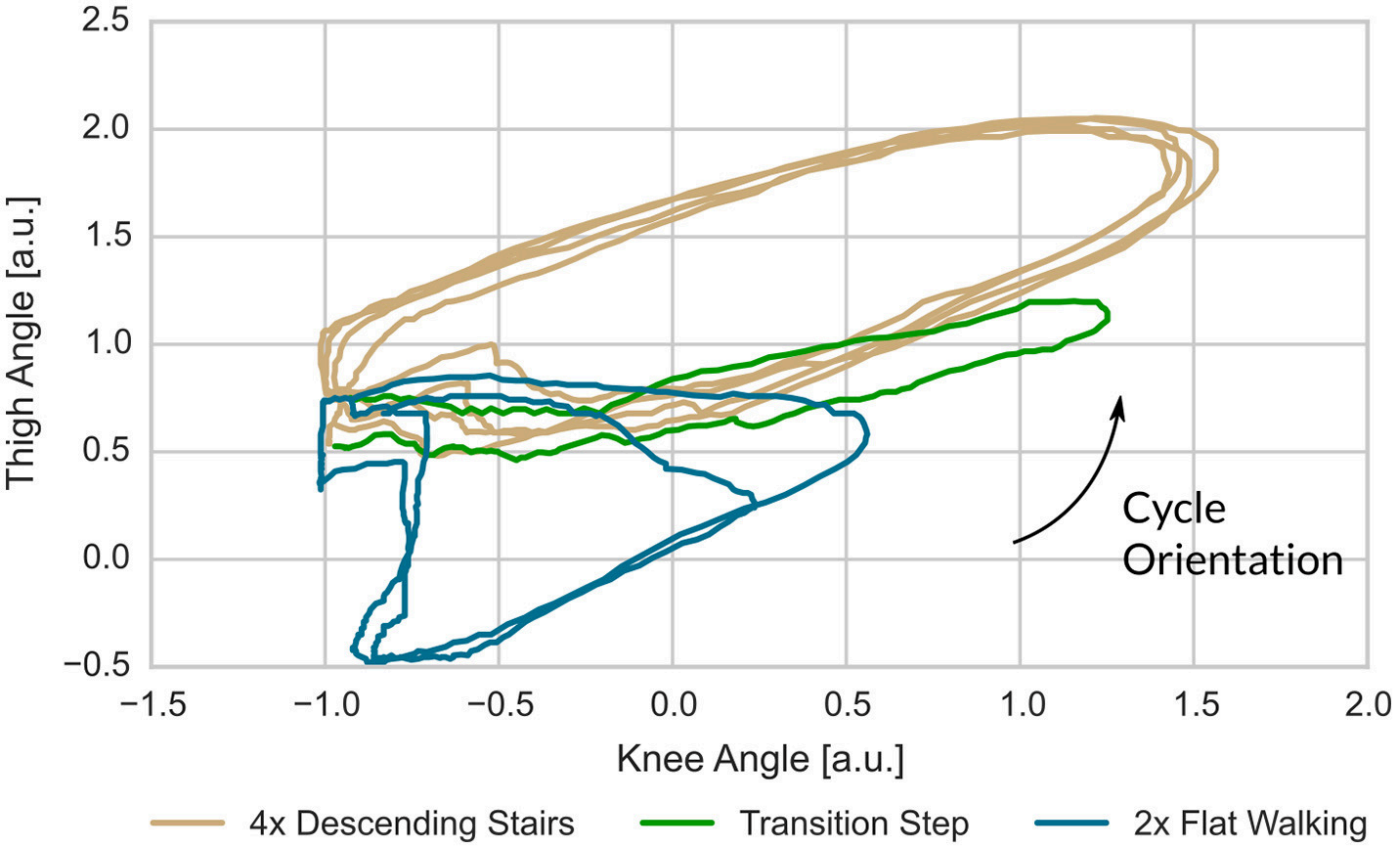
For the label mismatch of Figure 13, the plot of thigh- over knee-angle shows that the transition step has quite unique properties. Note, that the predicting gait models work on a history going back 0.2 s, i.e., they are working on a higher dimensional space and thus can easier separate these gaits.

The dynamics of knee and thigh angle (Figures 13, 14) show the transition step between descending stairs and walking on flat ground. It is easy to see that these steps are neither similar to one nor the other gait in 2D when plotting the angles over time, or thigh angle against knee angle. For the predicting models, which are working on a higher dimensional history, the dissimilarity is even more drastic. As a consequence, prediction errors are high for all models for this kind of gait transition step. It is to be expected, though, that many of these transitions fall into swing-phase transitions, where highly varying dynamics are possible and the actual control output is not that important for a device supporting mechanically.

## 4. Discussion

The presented neural mechanisms set out as an adaptive orthosis controller, empowering users to control device behavior.

### 4.1. Gait phase representation

We implemented a neural single-gait controller to *individualize* gait support in terms of (1) the patient's gait dynamics with learning from observation and (2) direct user feedback with an interface for tuning, placing the patient in the loop. The gait phase abstracts gait dynamics and thereby removes dependencies on remaining abilities, except the ability to initiate motion. Furthermore, the gait phase removes the time domain from the sensory inputs. Thus, it transparently supports gaits of different speeds and step lengths (Figure 10) as well as standing; it provides immediate reactions to regular and critical events like stumbling. Variability in the training set enables use in varying environments such that a level walking controller supports even ground as well as slopes of several degrees (up to ±15 ° were tested but not presented here).

The presented user feedback is a minimal implementation, which allows to define an arbitrary damping function *c* in sufficient detail and allows the user to adapt *c* at run-time. It allows the users to understand the controller behavior in an experimental way: this way the users can develop an intuition of how changes to *c* modify the controllers behavior. Furthermore, it simplifies the mapping from the gait phase to a valve position. Calibration and transformation are not necessary, as the user implicitly deals with these nonlinear operations. From the users' perspective, the user interface allows to define the level of support required. More important, their opinion is directly included in the controller's behavior. This inclusion of the patient's opinion concerns one of the top reasons for device abandonment see (Phillips and Zhao, 1993) and references therein.

Quantitative measurements verify instant reaction to motion, and high accuracy in timing and tracking of the patient's gait. We validated experimentally that the timing unit determines the heel-strike with high accuracy in the order of the sampling frequency. Furthermore, testing under the assumption that the recorded steps were ideally and steadily progressing, the timing unit was shown to approximate a linear progression of gait phase for trained gaits. Our generic approach of function approximation as representation for the control output provides intuitive tuning of the control output.

While the accuracy of gait phase tracking shows that gait models for quite different gaits can be learned, like flat ground walking and stair climbing, it also makes clear, that training leads to specialization of the feed-forward controller. To support movement in different environments, the controller has to be extended with controllers for multiple gaits in such a manner that free motion is possible.

### 4.2. Gait selection

Specialization to one gait in the single-gait controller is overcome by a gait selection process based on predicting models. To support a gait, the controller therefore needs (1) a timing module with training samples, (2) the desired output shaping module, and (3) a predicting model which can be trained with the same samples as (1). This modular control approach overcomes *design* problems which typically restrict supported motion and the patient *target group*.

Based on the internal models' prediction errors, the gait selection swiftly chooses a single gait controller with the best fitting dynamics. Eighty-four percent of the steps in our experiments were already correctly identified at heel-off, most of them as part of a sequence of the same gait. But, the ≈84 % steps include at least 50 % of the 72 transition steps. The ≈13 % steps, which are identified between heel-off and heel-strike, indicate that gait recognition has to perform continuous. Figures 13, 14 indicate that the gait dynamics is not bound to switch at any specific point and shows the flexibility and precision of the presented approach. For example, the initial step after standing phases is typically handled by the stair climbing module, which supports only vertical lift-off.

A fall-back controller, based on the ground contact sensing FSR, enables save operation in cases when gait dynamics are not matched by a model. The requirement for a fall-back controller is especially associated with transition steps, which often are singular events. The use of a history enables swift detection of changes in the motion. But at the same time, a gait change in the history will reduce the precision of predictions. Therefore, the history length not only determines the accuracy of gait prediction, but it also determines the frequency of changes, which can be tracked.

### 4.3. Advantages and limitations

The greatest advantages of the presented approach lie in (1) its flexibility, as only the equipped sensors determine which gaits can be differentiated, (2) its implicit support for stumbling support, due to ground contact directly shifting the gait phase toward stance phase, (3) device independence, and (4) independence of remaining abilities, as long as circular motion can be initiated.

A difficulty in the evaluation of the presented approach lies in the handling of transition steps. As gait transitions can seemingly happen anytime, training data will not cover them in all possible variations. This singular nature of transition steps was not captured in the user annotations. Nonetheless, the results show that the controller is able to choose a gait with similar dynamics in many cases. In these cases, the user's annotation describes an intention, but not necessarily provides the best match to gait dynamics. In other words, the annotations are only valid for steps without transitions, for which excellent recognition rates could be seen even with 100 Hz sampling rate and 3 channels. For transition steps, a broad selection of training data will allow to address many transitions. For all other cases, the fallback controller has to provide save operation, i.e., guarantee support in stance phase, which can be achieved with the FSRs.

A general problem is the question of the number of supported movements. While three gaits were sufficient to control all motions during experiment sessions, it is still unclear how many gaits need to be supported for comfortable operation in everyday life. At the same time, support for more gaits might fill gaps in gait transitions as more independent motions are supported.

The presented control approach integrates the user into the tuning process and allows to directly model individual movements. We believe that this approach improves the handling of *gait deviations* and *device acceptance*. Still, the presented experiments have been conducted with a healthy subject. Thus, patient tests have to be undertaken to understand the interaction and consequences for real patients.

For patient tests, the user interface should be simplified. Instead of defining the damping function via a set of function values over a grid of support points, more suitable parameters should be chosen. A promising idea would be to focus on the start and end points of the support periods. Considering these together with the amplitude and the slope should provide an interface which is easy to understand, but even easier to handle.

### 4.4. Gait phase tracking in the literature

Li et al. (2014) aim for a similar result by gait phase tracking on the contralateral leg. Inference of the controllers internal gait phase is based on the assumption of a constant phase shift to the ipsilateral leg. Besides practical issues with the instrumentation of the contralateral leg which directly impact comfort of use and visual appearance, it is important to note that constant phase shift can only be assumed in non-critical situations. Especially when stumbling or external forces disturb this phase shift, the contralateral leg does not reflect the device's state. The presented approach always faithfully reflects the ipsilateral state, keeping the procedure of device application to the ipsilateral leg.

The first prosthetic device to reduce its wearers energetic cost of walking were presented in Malcolm et al. (2013) and Mooney et al. (2014). The effect was highest, when device activation was triggered at ≈43 %. This result indicates that control based on gait progress presents an interesting approach to pursue.

### 4.5. Multi-gait support in the literature

Besides the here presented prediction error to invalidate gait models, many other approaches (Meyer, 1997; Mazzaro et al., 2005; Ding, 2008; Varol et al., 2008, 2010) are proposed. They are based on, for example, Gaussian mixture models or hidden Markov Models. Unfortunately, all of these studies discuss different selections of gaits, gait variations. For this reason, an actual performance comparison is difficult and would most likely be possible for image sequence based approaches (Mazzaro et al., 2005; Meyer, 1997), which are unfitting for prosthetic devices due to their outside-view on the walker. Furthermore, this study was working with a healthy walker. Still, average success rates between 83 and 94 % are comparable to vision based model invalidation approaches (Meyer, 1997; Mazzaro et al., 2005).

Besides image sequence based approaches, the literature mostly covers active prostheses. Due to space and weight limitations, active prostheses are of higher practical relevance than active orthoses, and therefore more present in the literature. Here, we will not cover technical differences, but focus on the controller.

Lawson et al. (2013) present a prosthesis controller for stair ascent and descent. The FSM architecture prevents the easy inclusion of other gaits and the missing support of level walking omits the region of high model overlap in this study. Sup et al. (2011) presents a hierarchy of FSMs, where one outer FSM with a slope estimator selects from three slope specific FSMs for 0, 5, and 10 °, respectively. While the fixation to slopes is incompatible with the gaits of this study, the addition of parameter estimation would provide beneficial input to the presented controller. A history based Gaussian mixture model differentiates standing and walking in Varol et al. (2008), selecting gait-specific FSM controllers on the fly. This study is based on seven signals sampled at 1, 000 Hz. An offline analysis was performed to reduce the dimensionality of the input for the Gaussian mixture models. In another step, the history length was increased, until the method provided a 100 % success rate. History frames of 50, 100, 200, or 400 samples were tried and finally a window size of 100 samples was selected with an overall delay of 430 ms. Later, this approach was extended to include sitting motion (Varol et al., 2010). The selection of sensors was described as task specific. In this study, the optimal delay was 500 ms.

All in all, the cross section of literature shows unique, and often incomparable gait selections and approaches. A similar approach with instantaneous selection was used in Varol et al. (2008, 2010) to differentiate standing, walking, and sitting motion. In contrast, the dynamics based gait tracking in our approach renders the recognition of standing superfluous. This focus on the device configuration simplifies data processing and needs neither explicit models of the device or gait nor expensive preprocessing. The presented work is based on only 3 sensors sampled at 100 Hz. Further improvements can be implemented with estimators of environmental parameters, sensors which provide differentiating inputs, or higher sampling frequencies. Especially with higher sampling frequencies, extensive optimization of the history and controller parameters, like amplification gains and weights could lead to significant improvements.

In comparison to biologically inspired modeling of modular motor learning and control, our control mechanism implements a partial function of the internal models for motor control proposed by Wolpert and Kawato (1998). The internal models are classified into three types: inverse internal model (the system calculates a motor command from a desired trajectory/state information), forward internal model (the system predicts sensory consequences from efference copies), and integrated internal model (the system integrates both inverse and forward models). In our case here, our shaping module acts as an inverse internal model that translates a user desired damping curve (i.e., desired trajectory) into a proper valve control command (i.e., motor command).

### 4.6. Outlook

Further optimization is possible with the many parameters in prediction, gait selection. Here, also other machine learning techniques can be employed (e.g., self-organizing learning of an adaptive resonance model Grossberg, 1987) is possible. The application of additional sensors, like torque sensors in the joints or IMUs, can improve differentiation.

Patients tests can show if the desired aims can be reached with the presented approach in real-world scenarios. Therefore, they are very important for future research.

The most interesting aspect is that our approach provides the building blocks for a completely self-learning controller. We demonstrated generalization of gait patterns, adaptation to changes in gait and in the environment as observed via gait changes. The user interface allows a user to adapt the support to individual needs. Still, at the stage presented here, the controller is not fully adaptive to a user in that it neither 1. automatically updates gait patterns, 2. damping output (lifetime of adjustments), nor does it learn new gaits on its own. Nonetheless, the modular structure allows to pursue these advanced aims. Additionally, other procedures (e.g., reinforcement learning or imitation learning) can be employed for offline training, where the subject provides the reward (good or bad) according to a given profile, or for fitting to the pattern of damping in human walking (Nakanishi et al., 2004).

Observation based training can be implemented at runtime, constructing and improving gait models continuously. A simple approach is to continuously add new samples to the training set and update the multi-layer perceptron networks's weights. The classification of recorded steps can be used to create new models, when new observations contradict existing models. In this way, bootstrapping of the controller can consist of a mostly generic model for walking on flat ground and an appropriate fallback controller. Then, during everyday usage, the controller adapts to the patient and vice-versa, while the patient can always influence the control output. Suggestions for automatic tuning could be generated and tested in accordance with the patient, based on gait quality assessment in the controller. In this way, patients would be empowered to fit their own orthosis, hopefully improving trust into and the general opinion of the device.

## Ethics statement

The experiment was performed in accordance with the ethical standards laid down by the 1964 Declaration of Helsinki. We followed the relevant guidelines of the Germany Psychological Society according to which this experiment, given the conditions explained above, does not need explicit approval by an Ethics Committee (Document: 28.09.2004 DPG: Revision der auf die Forschung bezogenen ethischen Richtlinien).

## Author contributions

J-MB, FW, and PM designed the research. J-MB developed the neural mechanisms. J-MB and PM carried out the experiments. J-MB and PM analyzed data. All authors wrote and reviewed the manuscript.

### Conflict of interest statement

The authors declare that the research was conducted in the absence of any commercial or financial relationships that could be construed as a potential conflict of interest.

## Footnotes

^1^If we consider, for example, Figures 13, 14, they show a transition from descending stairs to flat walking. We see that after the heel strike follows a period, where the dynamics seem to mostly follow the stair regime. This is followed by a period where differences to both gaits occur. Here, it is difficult to determine the gait until after the maximum knee flexion, when the gait dynamics converge to those of the flat walking regime. As the flat walking gait is only reached during the extension of the knee, we assume that the new gait is reached during the swing phase. Still, the transition changes the whole step's dynamics.
